# Fog Fragment Cooperation on Bandwidth Management Based on Reinforcement Learning

**DOI:** 10.3390/s20236942

**Published:** 2020-12-04

**Authors:** Motahareh Mobasheri, Yangwoo Kim, Woongsup Kim

**Affiliations:** Information and Communication Engineering Department, Dongguk University, Seoul 04620, Korea; mm.mobasheri@dongguk.edu (M.M.); woongsup@dongguk.edu (W.K.)

**Keywords:** Internet of Things, fog computing, reinforcement learning, Q-learning

## Abstract

The term big data has emerged in network concepts since the Internet of Things (IoT) made data generation faster through various smart environments. In contrast, bandwidth improvement has been slower; therefore, it has become a bottleneck, creating the need to solve bandwidth constraints. Over time, due to smart environment extensions and the increasing number of IoT devices, the number of fog nodes has increased. In this study, we introduce fog fragment computing in contrast to conventional fog computing. We address bandwidth management using fog nodes and their cooperation to overcome the extra required bandwidth for IoT devices with emergencies and bandwidth limitations. We formulate the decision-making problem of the fog nodes using a reinforcement learning approach and develop a Q-learning algorithm to achieve efficient decisions by forcing the fog nodes to help each other under special conditions. To the best of our knowledge, there has been no research with this objective thus far. Therefore, we compare this study with another scenario that considers a single fog node to show that our new extended method performs considerably better.

## 1. Introduction

Since the emergence of the Internet of Things (IoT), the number of devices increases faster [1]. As more devices create additional data, the new term big data was coined with three principal characteristics [2,3,4]: volume, velocity, and variety. The Cisco Visual Networking Index Complete Forecast predicted that 26.3 billion such nodes would be connected to the Internet by 2020 [5]. As a result of these developments, network bandwidth and latency issues have become more significant. To prevent long latencies, data loss, and the subsequent wrong decisions caused by bandwidth limitations, it is not logical to transfer all data to the cloud when, at every moment, devices produce numerous data. Therefore, fog computing has emerged as a complement to the cloud to handle distributed processing, networking, and storage resources. Due to the proximity of fog devices to end users, they can adequately support applications with quality of service (QoS) and real-time constraints, especially in emergencies.

If data are analyzed in the cloud layer, the delay caused by multiple layers of network delivery increases. The ideal approach is to make all operational decisions as soon as data can be turned into a meaningful context. Contextual understanding of data helps systems make faster and better decisions. If fog devices use machine learning (ML) approaches, their intelligence fulfills several aspects such as self-learning solutions; therefore, decisions can be made more quickly with less communication with cloud servers [6].

The traffic control architecture of [7], based on the fog computing paradigm, is proposed through the reinforcement learning (RL) approach to avoid a traffic jam and overcome the communication bandwidth limitations and local optimal traffic control flow. This architecture only considers the bandwidth limitations between the fog node and cloud, and vehicles (IoT devices) can communicate with the fog node without this limitation. Moreover, the smart environment is limited to smart city traffic; therefore, there is a single fog node. The importance of the smart network performance in the QoS is discussed in [8], which states that any wireless communication technology performance depends first on the bandwidth and latency factors. The limitations to revise the IoT architecture and user requirements of multimedia data are specified in [9]. As computation is the key required feature for multimedia IoT, cloud computing limitations are identified in exhausting the bandwidth and energy-constrained networks. This article also discusses the use of ML as a critical aspect for the feature extraction of meaningful information from unstructured multimedia data. In addition, this paper presents various event-processing approaches to decrease the network overhead and latency. Due to bandwidth wastage and latency in IoT applications where nodes have adaptive data traffic, a new superframe structure that is compatible with the existing parameters of the wireless standard is proposed in [10].

Du et al. [11] use a fair algorithm to minimize the maximum cost among all end devices and considers a decision-maker in the fog node, which receives offloading requests from end devices. According to the collected offloading requests of all users and the rapid wireless channel gains, it performs optimization to decide where the applications should be processed (i.e., in the device locally, the fog, or the cloud). Jošilo and Dán [12] propose a game-theoretical model of the completion time minimization problem in which devices can either perform their computations locally or offload them to a nearby device or cloud. Zhou et al. [13] introduce a delay-optimized computation offloading problem in vehicular fog nodes. Zhao et al. [14] propose fairness scheduling metrics in which the end user should select multiple fog nodes considering the time-energy efficiency and priority of each fog node, and then offload different parts of the task to the nodes. Zhao et al. [15] propose an online low-complexity fog-enabled multitier operation scheduling algorithm that optimizes node assignments jointly at the control tier and resource allocations at the access tier. Ali et al. [16] propose an optimization problem, where both fog nodes and IoT devices make preference lists based on their workload and latency constraints. In contrast to many studies, where some have considered computation offloading to manage the network bandwidth, our aim is not to assign resource-intensive computational tasks to an external platform. In this study, our focus is on avoiding the delay caused by the bandwidth limitation using the current infrastructure.

During the last few years, many traditional environments have become smart, and the continuously increasing number of variant IoT devices with different requirements makes managing them difficult. This issue increases the number of fog devices. Moreover, environments are developing over time, and all have their own specific properties and conditions. Therefore, a framework is needed to fulfill the cooperation of fog nodes in emergencies. All fog nodes belong to the same smart city, as an example of a vast smart environment. They use a shared limited network backbone bandwidth for communication with each other and their connected IoT devices. Considering this network, defining the rules and providing supervision services are time-consuming and costly with the probability of error. The tendency toward automation is increasing over time, requiring the ability to adjust the bandwidth automatically. The ML approaches are suitable for achieving this goal. Among the various ML approaches, RL is a powerful option that does not require a supervisor or trainer to solve problems.

Mobasheri et al. [17] consider a small smart environment with only one fog node as an RL agent. This fog node needs more data in emergencies for further analysis of events, and it is responsible for managing a limited bandwidth among several connected IoT devices. The proposed RL approach enables the fog node to learn how to prepare the extra needed bandwidth for emergency devices, while the total limited given bandwidth of the network is not violated. This procedure is conducted through trial and error and by receiving consequent reward values. The fog node learns the optimal policy based on the received reward values without a supervisor.

All studies about fog computing including [17] have considered an integrated space for modeling the fog. In other words, fog is assumed to be an integrated cloud near the edge of the network. In contrast, we consider a disintegrated fog in a vast smart city that includs several fog nodes. We consider each fog node and its connected IoT devices as a fog fragment (scope). Therefore, the fog is divided into several parts or fragments with the same decision-making problem. Each fragment fog node is responsible for receiving information from the connected IoT devices. In emergency cases, the fog node prepares extra bandwidth to receive more data than usual for better quality for further analysis. Through these bandwidth increments, the total network bandwidth should remain fixed. Therefore, the fog node increases the bandwidth for needy devices by decreasing the bandwidth of devices that are not in an emergency. Moreover, through the proposed learning procedure, all fog nodes are forced to cooperate in lending extra needed bandwidth to neighboring fog nodes during emergencies under special conditions and return the bandwidth when the emergency is over. We usethe same technique, RL, to solve this cooperation problem. All fog nodes act as RL agents and learn the environment states to maximize the average received rewards. This cooperation improves performance, especially in terms of convergence time.

The remainder of the paper is organized as follows. The research problem is defined in detail in Section 2. In Section 3, we formulate the decision-making problem and present the proposed method. Then, we describe the simulation of the proposed approach in Section 4, followed by the conclusions in Section 5.

## 2. Problem Definition

In this paper, we consider the decision-making problem of one fog node and extend it to a vast smart environment with several fog nodes that cooperate. First, we consider a smart fragment in an environment with a single fog node connected to several IoT devices, specified via different priorities. The total bandwidth of the environment network is fixed and limited; therefore, the allowed amount of bandwidth for each device is predefined. These devices experience normal situations and emergencies. When a device faces an unusual situation, it is responsible for informing its connected fog node and obtaining permission to send more data. Therefore, the fog node can receive a better analysis report for higher levels of its hierarchical management system and receive subsequent higher-level decisions.

As sending more data requires more bandwidth, the device must violate its assigned bandwidth amount. As the network bandwidth is limited, the fog node is responsible for learning the best selections among devices in ordinary situations to help devices in emergencies by decreasing their transmission rates to the fog node. The fog node acts as an RL agent; therefore, its learning is reinforced over time. After learning convergence, the fog node decisions become optimal and oriented toward the future; that is, the fog node selections help the system meet the minimum emergencies or minimum number of devices with a lack of bandwidth by helping others. The RL fog node gains the optimal policy, although it does not have a supervisor or information about the distribution parameters of future emergencies.

The smart environment management system fixes and predefines the amount of allowed primary device bandwidth for transferring data to the connected fog node, the amount of required additional bandwidth in emergencies, and the priorities based on their locations or responsibilities. Moreover, devices are categorized into three types: lowest priority, highest priority, and those between these extremes. The first type of device never have an emergency and are always candidates for helping emergency devices if they have sufficient bandwidth. The second type of device never helps emergency devices because they are the most important devices in the network and should always use their full bandwidth capacities. The third type of device can help devices in emergencies if they are not also in an emergency and have sufficient bandwidth.

A feasible device (helping candidate) set including the lowest and middle priority devices with normal situations and enough available bandwidth is created in each time step for the current emergency devices. In an emergency, the fog node must select a device from the feasible device set and decrease its bandwidth to increase the bandwidth of the needy device for further communication. Therefore, the goal of the fog node is to learn the best helper from the feasible device set based on the device priorities because when there is a helper with a lower priority, choosing a higher-priority helper is not efficient.

**Figure 1:**
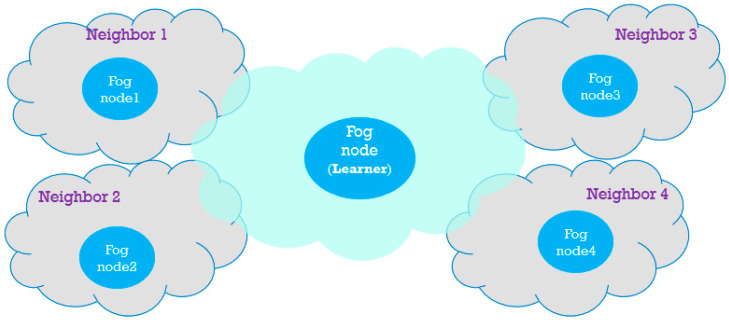
Five instances of fog fragments in a neighborhood.

The scenario assumed in [17] is simple and suitable for a smart environment that does not require more than one fog node. As the smart environment becomes more extensive, the number of fog nodes increases. Today, smart cities, as an example of large smart environments, include many smaller smart environments such as smart homes, smart factories, smart transportation, and smart health care, which all have related fog nodes. Moreover, the number of these smart environments increases over time while their continuous development increases. As the total limited bandwidth is shared among these fog nodes and their connected IoT devices, the cooperation of these fog nodes is required. In this paper, we do not consider an integrated fog infrastructure because of the consideration of several smart environments in the smart city. Instead, we assume a fog fragment for each smart environment, as illustrated in Figure 1. Therefore, we can consider each fog node and its connected IoT devices as a fog fragment and consider the cooperation of these fragments in using the same limited bandwidth. Each fog fragment works the same, as explained at the beginning of this section. Moreover, fog nodes are responsible for helping their neighboring fog nodes under a defined condition. Although all fog nodes are responsible for learning, we mention one of them as the learner to specify it from others in order to focus on one of the fragment’s internal operation.

Consequently, each fog node should learn the condition of receiving help from its neighboring nodes aside of learning the best decisions about helping the connected emergency devices. For simplicity, we assume that each fragment requires one fog node and that the devices belonging to this fragment are connected to this single fog node. The fog node selection problem by IoT devices is not considered in this study.

At the beginning of each time step, every fog node introduces its lowest-priority devices with the current bandwidth to the neighboring fog nodes as a feasible help list. If an emergency occurs at one of the fog fragments, it can obtain help from one of the neighboring feasible lowest-priority helpers under one condition, using the neighbors’ received feasible help lists, and it should inform the neighboring fog node about the selected helper and its bandwidth after helping. Therefore, this fog node can keep its fragment performance high and prepare the required extra bandwidth for emergency IoT devices to receive sufficient data for further analyses of the events causing the emergencies. The conditions that allow fog nodes to obtain help from others are defined at the beginning of smart city life. The condition that we consider is that, if no feasible device with a priority under a defined threshold is in one of the smart city fragments with an emergency event, its fog node can use the neighbors’ help list and select a neighboring helper with sufficient bandwidth for the current emergency device. The threshold is predefined at the beginning of the smart city life by smart city management to determine the limits of obtaining help from neighboring helpers mentioned in the received help lists. Therefore, the fog node is responsible for selecting a helper from its own fragment when a device exists in its own feasible device set with a lower priority than the threshold. Otherwise, the fog node can select a helper from other fragments in its vicinity. If the threshold is set to the highest priority, the fog node should never obtain help from neighbors, and if it is set to the lowest priority and no lowest-priority devices are in the feasible device set, the fog node obtains help from neighbors even in cases in which there are some feasible devices with higher priorities. Therefore, the threshold should be appropriately defined to ensure a logical tradeoff between selecting from its own or neighboring feasible devices.

**Figure 2:**
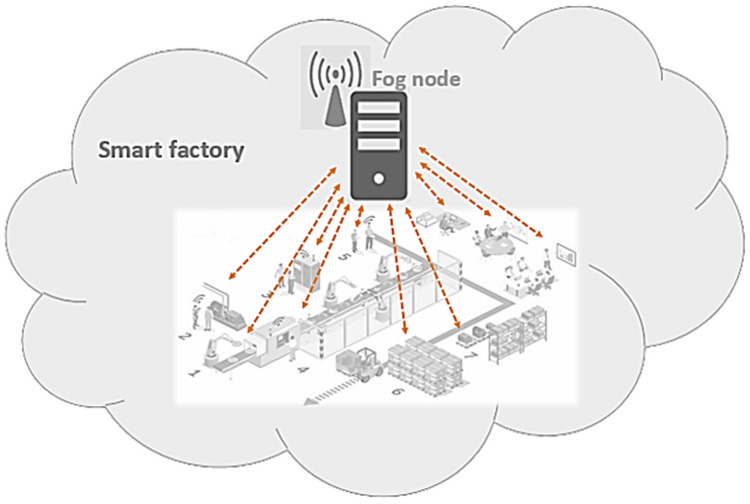
Smart factory as a smart city fragment.

Two reasons can cause a smart environment to not have a feasible device with a lower priority than the defined threshold for helping emergency devices: (1) the sum of the required extra bandwidth of several emergency devices in a fragment can be more than the sum of the current bandwidth of its feasible devices that have lower priorities than the threshold; and (2) all devices in a fragment have high priorities and should always be active such as in a smart factory. For example, a significant event occurs in the smart factory in Figure 2 and involves high-priority devices. Considering the bandwidth limitations of the communication lines [18], what happens when several sensors have emergencies when all of the factory sensors must be in active mode? In this case, the fog node requires more data with better quality for further decisions, so it asks the corresponding sensors to increase their transferring data frequency. As transferring more data requires more bandwidth, the fog node selects appropriate helpers among the neighbors’ feasible help list and borrows their bandwidth in the amount required for the emergency devices. Then, it informs the neighbors about these loans. The assignment of extra bandwidth becomes complicated when the number of needy devices increases. This issue considerably affects the environment when saving a millisecond is essential to prevent damage and failure.

We consider a dedicated wireless interface for the fog node cooperation, that is, fog node communication operates at different frequencies than those used for fog node communication with the connected IoT devices.

Based on the RL approach, each fog node tries all feasible helpers one by one among the neighbors’ feasible help list and the feasible device set in each visit of every state, without considering the conditions for obtaining help from the neighbors. In other words, the fog node is free to experience all feasible devices among its feasible device set and neighbors’ help list. In return for every selection, the fog node receives a reward value that indicates the quality of its recently selected device as a helper. In this way, the fog node learns the best helpers during the learning process through the received rewards and uses the best experience for successive similar future visits without a supervisor.

A significant issue is that defining the exact rules is not a proper approach. We should encourage or punish the fog node as an RL agent to learn optimal conditions to either obtain help from others or use its own feasible helpers. Therefore, even using the neighbors’ helpers should be learned over time by the fog nodes without instructions from a supervisor. In an RL approach, the most important part is defining the reward function, and the agent can understand how its actions affect the environment through this function. This paper only focuses on the bandwidth limitation and not on eliminating occurred emergencies because higher-level decisions require more information and, consequently, more bandwidth.

## 3. Reinforcement Learning Modeling

As already mentioned, several fog fragments are assessed in this study. To explain their operations, we focus on the operation of one fog node in its fragment. To specify it from its neighbors, we call it the learner fog node or just “the learner”. All other fog nodes have the same operation as this learner. We model this learner’s decision-making problem concerning selecting the best device as a helper in an emergency among its own and its neighbors’ feasible lowest-priority devices in a way that the received reward value is maximized, whereas the received punishment value is minimized. For this purpose, the state, action, and reward functions are defined as follows.

### 3.1. State

The state array, *flag*, presents situations for all of the *n_d_* connected devices to the learner, numbered from 1 to *n_d_*, in the time step *n*. Each element of *flag* indicates the situation of a connected device to the learner based on its value. It can be 0 to show that the related device is in a normal situation or 1 to indicate an emergency. In every time step, the learner updates *flag* based on the emergency reports from devices that have emergencies. As the emergency device situations become normal, the related indices of *flag* are updated from 1 to 0.

### 3.2. Action

Action selections and learning processes are made only in emergencies in which the learner should perform appropriate actions to prepare the sufficient required bandwidth for the needy devices. The learner determines emergencies by searching for elements of the *flag* array that recently changed to 1 at the beginning of each time step. If an event has happened in the area of device *i* (*d_i_*), the learner is responsible for selecting a device (*d_k_*) from the feasible device set and assigns a bandwidth of *d_k_* to *d_i_* as needed. As mentioned, this set contains IoT devices with different priorities (except the highest) in normal situations (∀ *d_k_* ∈ feasible device set: *flag*(*k*) = 0) with sufficient bandwidth for helping *d_i_*. Action *a_i_* shows *k*, which implies that a device with index *k* is chosen for helping the emergency device with index *i*. For this purpose, various arrays and matrices are needed, which are defined as follows:

- According to their indices at the beginning of the smart city network, array *b_p_* holds the bandwidth of all devices, which is predefined through the city management system.
- Array *b_c_* denotes the current bandwidth of all devices according to their indices.
- Array *b_r_* represents the sufficient required bandwidth of each device for emergencies.
- Array *p* indicates all device priorities designed by the city management system.
- If an emergency occurs for *d_i_*, element (*i*, *k*) of matrix *aid* is changed from 0 to 1 to denote that device *k* helped device *i*.
- When element (*n*, *i*) of matrix *emg* changes to *b_r_*(*i*), it indicates that *d_i_* encountered an emergency in time step *n* and required additional bandwidth (*b_r_*(*i*)).
- The Boolean array *track* contains *n_d_* elements, and elements with a value of 1 indicate devices that have received help and are still in an emergency.

In the RL approach [19,20], an agent selects an action in two ways, randomly or based on experience. At the beginning of the learning process, the probability of random action selection is higher than that of selecting an action based on experience as the learner does not have any experience in the environment. Thus, it selects actions randomly, observes the results, and strengthens its experience step by step. Over time, as the learning progresses, and the learner becomes more of an expert, the probability of random action selection decreases. Equation (1) expresses the probability of a random action selection among all feasible actions in time step *n*:

(1) $$\varepsilon =n^{-0.015}$$

where *ɛ* should be optionally defined to offer the learner the opportunity to explore all feasible actions in every state. Then, *ɛ* becomes smaller step by step to allow the learner to use its experience more than before, as one of the basics of the RL approach [19]. With the possibility of 1 − *ɛ*, an action is selected according to Equation (2):

(2) $$a_{i}=argmax_{k\in feasible}\left(Q\left(i,k\right)\right)$$

Therefore, the action with the maximum Q-value is selected according to the previous visits of the same state and different experienced feasible actions. The learner is responsible for assigning the required additional bandwidth to the needy devices and taking the borrowed bandwidth back to the resources when needy devices are no longer in an emergency. In other words, when the needy device *d_i_* sends a report about its emergency to the learner, the learner selects a helper (*d_k_*) based on its strategy and assigns a certain amount of bandwidth to *d_i_* according to *b_r_*(*i*). Then, it changes *track*(*i*) to 1. Finally, when *d_i_* returns to its normal situation, the learner applies three changes. First, it sets the related element of *flag* and *track* arrays (*flag*(*i*) and *track*(*i*)) from 1 to 0. Second, it changes *aid*(*i*, *k*) from 1 to 0. Third, it returns the additional assigned bandwidth from *d_i_* to *d_k_* (line 14 of the Algorithm 1).

### 3.3. Reward Function

By performing every decided action, the learner receives a reward value that helps it understand its decision quality. In general, assigning sufficient bandwidth to a needy device results in a reward increase. To define the reward function based on the above explanation, we formulated *fnort* as follows (Equation (3)):

(3) fnort=ʘ(flag,track)

where the symbol ʘ represents the *XNOR* gate. Therefore, *fnort* becomes 1 only when the learner has helped a needy device or both related elements in *flag* and *track* are 0. When a device requires help but the learner has not helped it, the value of the related index of *fnort* remains 0.

As mentioned, one issue that causes an action selection to become optimal is selecting a device with the lowest priority of all feasible devices that can help. Therefore, this constraint should affect the reward function. For this purpose, our main algorithm forms two more arrays. Array *dp* indicates the number of devices with the same priorities in the learner fragment regardless of whether these devices are in the feasible device set. For example, *dp*(1) = *x* means that *x* devices have a priority value of 1 in the learner fragment. Then, the *ddp* array is formed using the *dp* array in which the *i*th element of this array indicates the number of devices in the learner fragment with a lower priority than *i*. Using the *ddp* array in Equation (4), *punish*(*i*) should be calculated to indicate the number of helpers in the feasible device set whose priorities are lower than the priority of the selected helper (*d_k_*) for needy device *i*:

(4) $$punish\left(i\right)=length\;\left(\mathrm{feasible}\;\mathrm{device}\;\mathrm{set}\right)\;:\;p{\left(j\right)}_{\;\;d_{j}\;\in \;\mathrm{feasible}\;\mathrm{device}\;\mathrm{set}\;}\leq \;p\left(k\right)$$

Another factor called the *penalty* affects the reward function only when no device has the helping conditions because of the poor operation of the learner. Therefore, the reward value decreases by the *penalty* to alert the learner. Moreover, this alarm should be sufficiently high to be a good punishment for the learner. Equation (5) assigns the number of all devices with lower priorities than the priority of the needy device as the *penalty* (the priority of device *i* (*d_i_*) is *p*(*i*)):

(5) penalty =ddp(p(i))

Moreover, the reward function should be defined to give the learner a higher reward value when it selects a connected feasible device as a helper, which has a lower priority than the defined threshold, rather than those in the neighbors’ help list. In other words, when the learner selects one of the neighbors’ feasible devices mentioned in the help list when at least one feasible device is in its own fragment with a lower priority than the threshold, it should receive a value called *blame* as a punishment. Equation (6) counts the number of devices in the learner’s feasible device set whose priorities are lower than the threshold.

(6) $$blame=length\;\left(\mathrm{feasible}\;\mathrm{device}\;\mathrm{set}\right):\;p{\left(j\right)}_{\;\;d_{j}\;\in \;\mathrm{feasible}\;\mathrm{device}\;\mathrm{set}\;}\leq \;\mathrm{threshold}$$

In this study, the reward function indicates the number of needy devices that have already received the required additional bandwidth plus the number of devices with normal situations, as the learner’s operation is one of the reasons for being in a normal situation, minus the first, second, and third punishment values as follows:

1. If no device exists with the helping conditions in the learner fragment because of its poor operation in the past, the first punishment value, *penalty*, decreases the reward value by the number of all the devices with lower priorities than the priority of the needy device in the learner fragment, not just those in the feasible device set.
2. If the learner selects a helper among its own feasible devices, the second punishment value, *punish*(*i*), is the number of helpers in the feasible device set whose priorities are lower than the threshold and priority of the selected helper for the current needy device (*d_i_*).
3. If the learner selects a neighbor’s helper, the third punishment value, *blame*, is equal to the number of devices in the learner’s feasible device set and has lower priorities than the threshold.

The learner does not receive a reward value in every time step *n* because some time steps do not require action selection by the learner. In other words, no new emergency has occurred for any of the smart city devices. Therefore, the learner receives reward values only in cases requiring action selection (selecting devices as helpers) among the feasible devices. Therefore, instead of counter *n*, we use *n_Q_* to represent the number of times the learner performs the Q-learning procedure and receives rewards. Finally, the *R_nQ_* function showing the reward is defined in Equation (7):

(7) $$R_{nQ}=length\left(find\left(fnort\right)\right)-punish\left(i\right)-penalty-blame$$

where the result of the *find* function is the elements of *fnort* whose values are 1. Then, with the *length* function, the number of helped and normal-situation devices is obtained. Finally, after checking all *flag* elements, performing the proper actions, and receiving the rewards, the learner updates the Q matrix using the main equation of the Algorithm 2 [19]. Equation (8) presents this updating procedure of the Q matrix:

(8) $$Q\left(i,a_{i}\right)=\left(1-\propto \right)Q\left(i,a_{i}\right)+\propto \left(R_{nQ}+\gamma *\;max_{k^{\prime }\;\mathrm{is}\;\mathrm{feasible}}Q\left(i^{\prime },k^{\prime }\right)\right)$$

where *γ* is a constant discount factor. When *γ* is defined as closer to 0, the recently received reward is more important than the next-step reward. Furthermore, *α_n_* is defined in Equation (9), where *cnt* is the number of visits of the state in which *d_k_* (*k* = *a_i_*) helps the emergency device *d_i_*:

(9) $$\propto_{nQ}=\frac{1}{cnt\left(i,a_{i}\right)}$$

### 3.4. Algorithm

Our main algorithm begins with the initialization of several parameters, matrices, and arrays. After initialization and before meeting the convergence conditions, the learner must check three conditions (lines 5, 10, and 14 of the Algorithm 1). The learner is responsible for changing the bandwidth of two devices only in the last two conditions. The second condition (line 10) is a status that denotes that a needy device is no longer in an emergency. Once the second condition is met, the learner must take the assigned extra bandwidth from this device (*d_i_*) and return it to the device (*d_u_*) that helped it. The third condition (line 4) checks the status of meeting the emergency conditions for device *i* (*d_i_*). If this condition is true, the learner selects a helper and decreases its bandwidth by the amount that the emergency device requires and then increases the bandwidth of the needy device. The helper selection is the action of the learner based on the Q-learning technique. Successful bandwidth management (*SBM*), which is defined in Equation (10), is the target function for evaluating the entire operation of the learner:

(10) $$SBM_{nQ}=length\left(find\left(fnort\right)\right)-countpun$$

where *countpun* calculates the total number of feasible devices whose priorities are lower than those of the selected helpers for all needy devices at time step *n*. This value is calculated via Equation (11):

(11) $$countpun=\underset{i}{\sum }punish\left(i\right).$$

**Algorithm 1** Main Algorithm1 2 3 4 5 6 7 8 9 10 11 12 13 14 15 16 17 18 19 20 21 22 23 24 Input:Initialize the priorities, primary bandwidth (*b_p_*), extra bandwidth in emergencies (*b_r_*),current bandwidth (*b_c_*) of all IoT devices, *flag*, time step (*n*), and Q matrix.While not converged, doIf all *flag* elements of the learner were 0 in the last two steps, do*countpu*n = 0Go to line 19 (2/4)ElseFor each element *i* of *flag* of the learner, doIf *flag(i)* is converted from 1 to 0, doFind device *u* that helped device *i* among all devices, including neighbors’helpers.Return the borrowed bandwidth from *i* to *u*Elseif *flag(i)* is converted from 0 to 1, doQ-learning algorithmEnd ifEnd forEnd ifMake a new *flag* for the next step for the learnerRepeat lines 5 to 19 for neighbors’ fog nodesCalculate *SBM_n_* using *countpun* considering all fragmentsCalculate the average of *SBM*Increase the time step *n* by 1End while**Algorithm 2** Q-learning Algorithm1 2 3 4 5 6 7 8 9 10 11 12 13 14 15 16 17 18 19 Make a feasible device set, including the nodes’ own feasible devices and the neighbors’ feasible help list.With probability *ɛ*, randomly choose device *k* among the feasible device setOtherwise, $a_{i}=\;argmax_{j\in \;\mathrm{feasible}}\left(Q\left(i,j\right)\right)$, where *j* is the index of the selected helper for helping device *i*If all devices were not able to help, doCalculate *penalty*ElseIf the selected helper is from its own devices, doCalculate *punish*(*i*)ElseCalculate *blame*(*i*)End ifEnd ifIncrease the bandwidth of device *i* and decrease the bandwidth of device *j*, as much as *i*
needsCalculate *R_nQ_* based on *punish(i), blame(i), penalty*, and the helped and normal devices.countpun = countpun + punish(i)Update Q matrix:$Q_{new}=\left(1-\alpha \right)Q_{old}\left(i,a_{i}\right)+\;\alpha \;\left(R_{nQ}+\;\gamma \left(max_{k^{\prime }\epsilon \;\mathrm{feasible}}\;\left(Q_{old}\left(i^{\prime },k^{\prime }\right)\right)\right)\right).$

## 4. Results

We consider a smart city including three fragments, with their IoT devices, which are all the same in bandwidth and priority. To investigate the fog fragment cooperation, in the first step, we consider the operation of a single fog node (the learner) that learns its own fragment states and makes decisions in emergencies about the best helpers among its connected devices and its two neighboring fragment devices mentioned in the received neighbors’ help lists, aside of helping its neighbors in their emergencies. Furthermore, we compared this scenario with the scenario in [17] in which no cooperation occurred among fragments where the fog node is isolated.

Although our considered fog node, the learner, can obtain help from neighbors and sense a better situation at first glance compared to the second scenario, first, the learner could not rely on the neighbors’ help because they are not always guaranteed to help their vicinity fragments. In some cases, it is probable that the neighbors’ lowest-priority devices do not have sufficient bandwidth for a connected emergency device of the learner. Second, the learner is responsible for helping its neighboring fragments with its own lowest-priority devices; therefore, managing its own emergency devices becomes harder. Third, the learner’s decision-making becomes more challenging because more options are available with different rewards.

**Figure 3:**
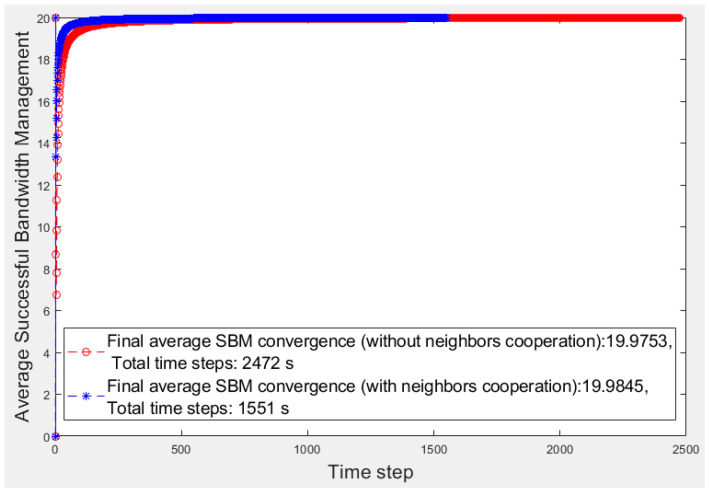
Final average successful bandwidth management *(SBM)* of the fog nodes without and with cooperation with neighbors.

**Figure 4:**
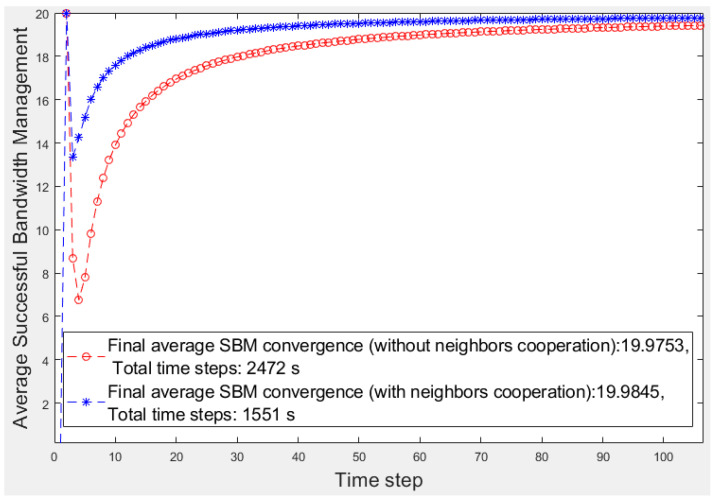
First 100 s of Figure 3.

In the first step, we focus on the learner’s fragment with 20 devices (*n_d_* = 20) that all sent video traffic to the learner. Each time step is considered to be 1 s. To simulate emergencies in each time step, the uniformly distributed pseudo-random integer function generates *n_d_* values randomly from {0, 1} for *n_d_* elements of the *flag* array to describe device situations in the learner’s fragment. When an element of *flag* becomes 1, the related device has encountered an emergency and needs help. The *flag* array, Q matrix, *ɛ,* and the threshold are initialized to 0, 0, *n^−^*^0.015^, and *n_d_*/3, respectively. The predefined priority array is input to the learning algorithm. For 20 devices, we consider six, four, and ten devices for Types 1, 2, and 3, respectively. Finally, we compar the learner operation with the fog node operation in the other scenario in which no cooperation occurred among fog fragments. Using the *SBM* average, we evaluate and compare two scenarios, one considering the fog fragment cooperation and the other considering isolated fragments. These scenario results are presented in Figure 3. Figure 4 displays a better view of Figure 3.

The red and blue curves denote the average *SBM* results of the isolated fog fragment and the learner fragment scenarios, respectively. Furthermore, the vertical axis denotes the average *SBM*, and the horizontal axis represents the time steps. Figure 3 reveals that the learner’s cooperation results converges to a higher average value of *SBM* (19.98) than the other results (19.97). Moreover, the total converging time steps of the learner’s learning (with an accuracy of 10^−5^) takes 1551 s, whereas the other scenario takes 2472 s. Every millisecond is essential in a smart environment. For a better analysis, in Figure 4, we focus on the first 100 s of Figure 3.

The number of devices in each fragment is 20 (*n_d_ =* 20); therefore, the highest ideal *SBM* value is 20. In the first time steps of learning, the number of emergencies is low, and the number of helping candidates is high; thus, both scenarios soon reach the highest *SBM*. As time continues and the number of emergencies increases, the number of feasible helpers decreases, resulting in the temporary descent of the average *SBM* values. As the learner has more options to select in emergencies because of its neighbors’ help, the operation is better than that of the fog node in the other scenario. Over time, the learning progresses, and the fog node experience strengthens; therefore, the fog node selections improve in both scenarios; thus, the average *SBM* of both algorithms increases. Finally, the learner converges to a higher average *SBM* than the fog node because it can obtain help from neighbors when no low-priority device or feasible device in its fragment is available because of its poor operation in the past.

After the convergence, the learning process for both fog nodes and the average *SBM* become stable. Once the learning process is complete, the learner can select the best helper among the helping candidates of its own feasible devices or neighbors’ help list based on its experiences according to the received rewards. Figure 3 presents the *SBM* of the fog nodes of two scenarios regardless of their neighbors’ *SBM*. In the following paragraphs, we investigate the proposed approach in a complete scenario in which the average *SBM* explains the average *SBM* of all fragments when they cooperate.

**Figure 5:**
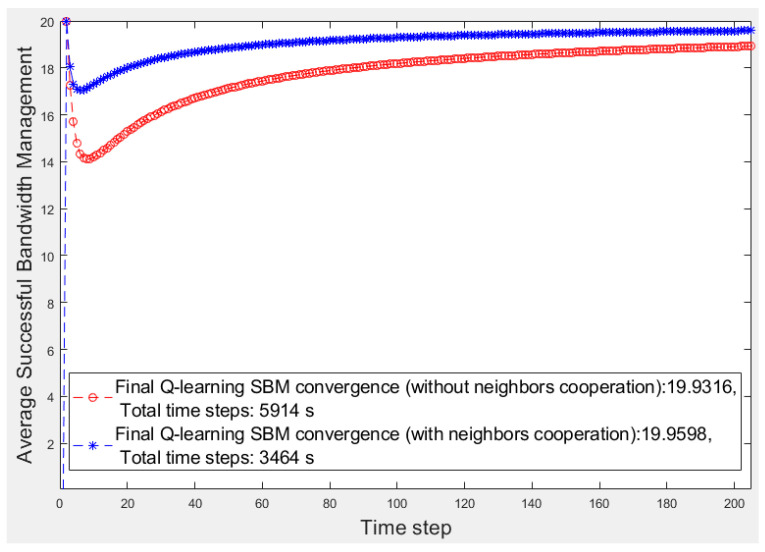
First 200 s of the average successful bandwidth management (*SBM*) of a three-fragment smart city network without and with fragment cooperation.

**Figure 6:**
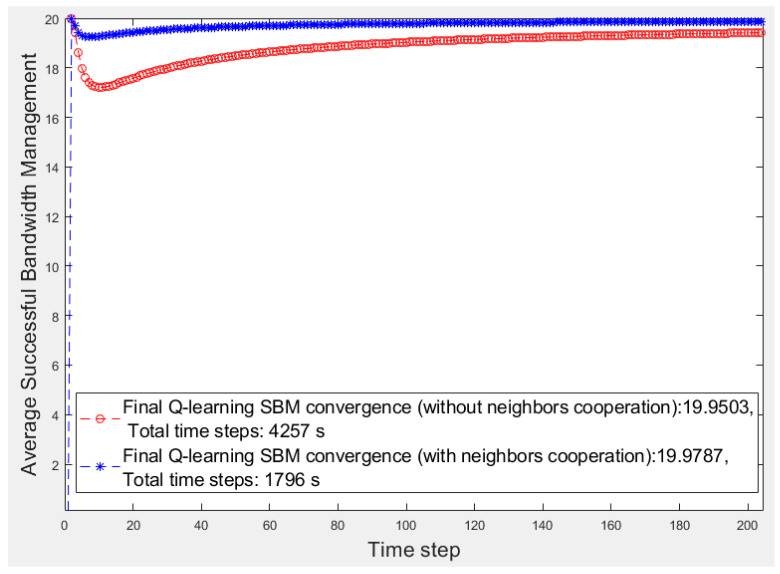
First 200 s of the average successful bandwidth management (*SBM*) of a five-fragment smart city network without and with fragment cooperation.

Figure 5 and Figure 6 present the first 200 s of two smart cities’ average *SBM* in which there are three and five smart fragments, respectively, and all are the same in terms of the number of devices and their priorities and bandwidth requirements. For a better comparison, Table 1 lists the results of all smart cities where w/o and w show the scenarios without and with fog fragment cooperation, respectively. We calculate the performance by obtaining the average *SBM* ratio and the elapsed time multiplied by 100. The superiority of the cooperation approach is apparent for both items of average *SBM* and elapsed time. Although the average *SBM* of the isolated fog fragments is close to the other, the periods have significant differences, whereas the time factor is critical in smart cities.

## 5. Conclusions

In a world where various environments are moving toward automation and being smart and where the improvement speed of equipment and number of users are exponential, management issues become more complicated and vital. Reinforcement learning as a ML approach can be suitable and helpful because it can adjust the system in its variable environment without supervisory instructions that have time and economic costs and the possibility of human errors.

This study considered a smart city as a smart environment with several smart fragments including some IoT devices using the same network with fixed and limited total bandwidth. Then, we proposed an RL approach to solve the bandwidth management issue in emergencies using fog nodes of smart fragments while forcing neighboring fog nodes to cooperate. Considering different fragments for the fog nodes, their cooperation resulted in better performance in less time. This cooperation can prevent the network failure of a fog fragment when the fragment cannot overcome emergencies. We compared this scenario with a scenario in which the fog nodes worked separately and demonstrated the power of the RL approach in the cooperation mode, especially in terms of convergence time, as saving milliseconds is essential to prevent damage and failure. Some proposed algorithm inputs are static such as device priorities and their extra needed bandwidth in emergencies. This approach applies to broader types of smart environments if these fixed inputs vary during fog node learning. In the next step, we will apply these improvements to a more general algorithm.

## Figures and Tables

**Table 1:** Average successful bandwidth management (*SBM*) results of the scenarios without (w/o) and with (w) the cooperation of fog fragments.

Figure No.	Average *SBM*		Elapsed Time (s)		Performance (%)	
	w/o	w	w/o	w	w/o	w
**3**	19.97	19.98	2472	1551	0.80	1.28
**5**	19.93	19.95	5914	3464	0.33	0.57
**6**	19.95	19.97	4257	1796	0.46	1.11

## Abbreviations

Parameter	Meaning
*n*	Entire system time steps
*nQ*	Learning time steps
*ɛ*	Probability of random selection
*α*	Learning coefficient
*γ*	Constant discount factor
*b_p_*	Predefined primary bandwidth of devices
*b_c_*	Current bandwidth of devices
*b_r_*	Predefined additional bandwidth required by devices
*p*	Predefined device priorities
*dp*	Its *i^th^* element shows the number of devices with priority *i*
*ddp*	Its *i^th^* element shows the number of devices with priority *i* or less than *i*
*punish*	Number of helpers in the feasible device set with lower priority than the current emergency device priority
*countpun*	Total number of helpers in the feasible device set with a priority less than that of the selected helpers for all needy devices in time step *n*
*penalty*	Number of all devices with lower priority than the current needy device priority
*blame*	The learner’s punishment when it chooses a neighboring device when some devices in its feasible device set have lower priority than the threshold
*emg*	When element *(n, i)* turns to *b_3_*, this parameter indicates whether an event has occurred for device *i* in time step *n* and whether it needs extra *b_3_* of the bandwidth
*aid*	When element *(i, k)* becomes 1, this parameter indicates that needy device *i* has received help from device *k*
*cnt*	*cnt(i, k)* denotes how many times device *k* was selected as a helper when device *i* went into an emergency state
*flag*	*flag(i) =* 0 indicates that device *i* is in the normal situation, and *flag(i) =* 1 indicates an emergency
*track*	*track(i) =* 1 indicates that device *i* has received extra bandwidth
*fnort*	*fnort(i) =* 0 indicates that needy device *i* has not received extra bandwidth
*a*	Selected action (helper)
*R*	Received reward for the fog node
*Q*	Element *(i, k)* represents the Q-value of the selected device *k* for helping device *i*
*SBM*	Successful bandwidth management
